# Rho and riboswitch-dependent regulations of *mntP* gene expression evade manganese and membrane toxicities

**DOI:** 10.1016/j.jbc.2024.107967

**Published:** 2024-11-05

**Authors:** Anand Prakash, Arunima Kalita, Kanika Bhardwaj, Rajesh Kumar Mishra, Debarghya Ghose, Gursharan Kaur, Neha Verma, Bibhusita Pani, Evgeny Nudler, Dipak Dutta

**Affiliations:** 1 Department of Molecular Biochemistry and Microbiology, CSIR Institute of Microbial Technology, Chandigarh, India; 2 Academy of Scientific and Innovative Research (AcSIR), Ghaziabad, Uttar Pradesh, India; 3 Department of Biochemistry and Molecular Pharmacology, New York University Grossman School of Medicine, New York, New York, USA; 4 Howard Hughes Medical Institute, NYU Langone Health, New York, New York, USA

**Keywords:** *Escherichia coli* (*E. coli*), gene regulation, transcription termination, manganese, membrane protein, riboswitch, translation, Rho, MntP, ROS

## Abstract

The trace metal ion manganese (Mn) in excess is toxic. Therefore, a small subset of factors tightly maintains its cellular level, among which an efflux protein MntP is the champion. Multiple transcriptional regulators and a manganese-dependent translational riboswitch regulate the MntP expression in *Escherichia coli*. As riboswitches are untranslated RNAs, they are often associated with the Rho-dependent transcription termination in bacteria. Here, performing *in vitro* transcription and *in vivo* reporter assays, we demonstrate that Rho efficiently terminates transcription at the *mntP* riboswitch region. Excess manganese activates the riboswitch, restoring the coupling between transcription and translation to evade Rho-dependent transcription termination partially. RT-PCR and Western blot experiments revealed that the deletion of the riboswitch abolishes Rho-dependent termination and thereby overexpresses MntP. Interestingly, deletion of the riboswitch also renders bacteria sensitive to manganese. This manganese sensitivity is linked with the overexpression of MntP. Further spot assays, confocal microscopy, and flow cytometry experiments revealed that the high level of MntP expression was responsible for slow growth, cell filamentation, and reactive oxygen species (ROS) production. We posit that manganese-dependent transcriptional activation of *mntP* in the absence of Rho-dependent termination leads to excessive MntP expression, a membrane protein, causing membrane protein toxicity. Thus, we show a regulatory role of Rho-dependent termination, which prevents membrane protein toxicity by limiting MntP expression.

Mn is an essential trace nutrient that regulates the functions of handful enzymes by serving as a cofactor or through differential metalation. Mn helps detoxify reactive oxygen species by acting as a cofactor of Mn-dependent superoxide dismutase and ribonucleotide reductase under iron starvation and oxidative stress (1, 2, 3). Mn also transiently replaces some enzymes' mononuclear iron cofactor, thereby restoring their functions under oxidative stress (4, 5). However, in excess, Mn inhibits cell growth and promotes cell filamentation by interfering with iron homeostasis in *Escherichia coli* (6, 7). Mn homeostasis in *E. coli* is mainly governed by the MntR transcription regulator. Under Mn shock, Mn-bound MntR represses *mntH* and activates *mntP*, which encodes Mn importer and exporter proteins, respectively (6, 8). MntR also represses *mntS*, which encodes a small peptide, to inhibit the MntP-dependent export of Mn (6, 8). It has been observed that the deletion of *mntP* makes *E. coli* cells highly sensitive to Mn (6, 7, 8). Several other mechanisms have also evolved to regulate the MntP levels in *E. coli*. For example, the expression of MntP is also regulated by Fur, a regulator that usually regulates iron homeostasis (6). The 5′-untranslated region (UTR) of the *mntP* forms an Mn-dependent riboswitch to upregulate *mntP* expression in *E. coli* (9, 10). All these reports suggest that the MntP-mediated export of Mn is crucial to handling Mn homeostasis.

The riboswitches usually modulate the expression of downstream genes when they are active in the presence of small metabolites (11, 12). Either they promote the formation of an intrinsic terminator inhibiting the transcription or act at the level of translation initiation, occluding the ribosome binding site (10, 11, 12, 13, 14). The *E. coli mntP* riboswitch belongs to the *yybP-ykoY* riboswitch family, which is named after the riboswitch-regulated *yybP* and *ykoY* genes of *Bacillus subtilis*, utilizes Mn as a ligand (10, 14, 15, 16, 17). Without Mn, the 229 nucleotides long 5ˊ-UTR of *mntP* maintains a “switched off” conformation of the riboswitch, making RBS inaccessible to the ribosome-mediated translation initiation (10). The presence of Mn stimulates alternate “switch-on” conformation, relieving RBS to interact with the ribosome freely (10). Our recent work has shown that the cellular alkaline pH favors the tight binding of Mn to the riboswitch to activate the latter. Mn activates an intrinsic alkalization circuit, overproducing cellular ammonia to attain this alkaline pH (9).

In contrast to *E. coli*, *B. subtilis*, and many pathogenic Gram-positive firmicutes require comparatively high levels of Mn (18). Manganese is essentially needed for the sporulation of *B. subtilis* (18, 19). To sustain their manganese-centric physiology, these organisms employ a wide range of homeostasis mechanisms. *B. subtilis* MntR mainly represses two different types of Mn uptake operons, one encodes a proton-coupled importer, MntH, and another encodes an ABC transporter (MntABCD) (20). Additionally, MntR activates the expression of MneP and MneS, two cation diffusion facilitator efflux pumps, that export Mn in necessity (21). Unlike *E. coli* MntP, the MneP and MneS exporters are not regulated by any riboswitches. However, the two genes, *yybP* and *ykoY*, which encode membrane proteins of unknown functions in *B. subtilis*, are regulated by Mn-dependent riboswitches (10, 14, 15).

The Rho helicase, a hexameric transcription termination factor, binds and threads the RNA through its central channel in a 5′ to 3′ direction. Once Rho recognizes a paused elongation complex (EC), it dissociates the RNA from the template DNA (22, 23, 24, 25, 26, 27, 28). Many auxiliary transcription factors can influence the Rho action. For example, elongation factor NusG binds both EC and Rho and stimulates termination ubiquitously (29, 30, 31). Rho function requires RNA sequences named Rho utilization (*rut*) sites, characterized by poorly conserved C-rich sequences with relatively little secondary structure (27, 32, 33, 34). Since riboswitches are the sufficiently long untranslated RNA portion of transcripts, they frequently act as platforms for Rho-dependent termination. The Mg^2+^-sensing *mgtA*-riboswitch in *Salmonella enterica*, FMN-sensing *ribB* and *ribM-*riboswitches in *E. coli* and *Corynebacterium glutamicum*, respectively (35, 36, 37, 38), lysine-sensing *lysC*-riboswitch, and thiamin pyrophosphate-sensing *thiB-*, *thiC-* and *thiM-*riboswitches from *E. coli* (39) are some examples where Rho-dependent termination is also reported. Despite thorough mechanistic studies, how the joint actions of riboswitch and a Rho-dependent terminator cater an evolutionary advantage to the bacteria to survive better is not addressed yet. The answer to whether the unhindered overexpression of the riboswitch-associated genes in the absence of Rho-dependent termination is detrimental to bacteria is missing.

In the present study, we show that apart from forming a Mn-dependent riboswitch, the 5′-UTR of *mntP* also serves as a platform for the Rho-dependent transcription termination to downregulate *mntP* expression. Mn exposure activated the riboswitch and sufficiently upregulated *mntP* expression by partially suppressing Rho-dependent termination to mitigate Mn stress. The Mn stress overexpressed MntP at a very high level in an *E. coli* strain devoid of the 5′-UTR. The cells exhibited slow growth, ROS production, and filamentation phenotypes. As these phenotypes fit perfectly with the context of membrane protein overexpression, we propose that Rho-dependent termination at the 5′-UTR presumably ensures standard membrane biology by suppressing the uncontrolled expression of MntP membrane protein.

## Results

### Inhibition of Rho function causes upregulation of *mntP* expression

**Figure 1:**
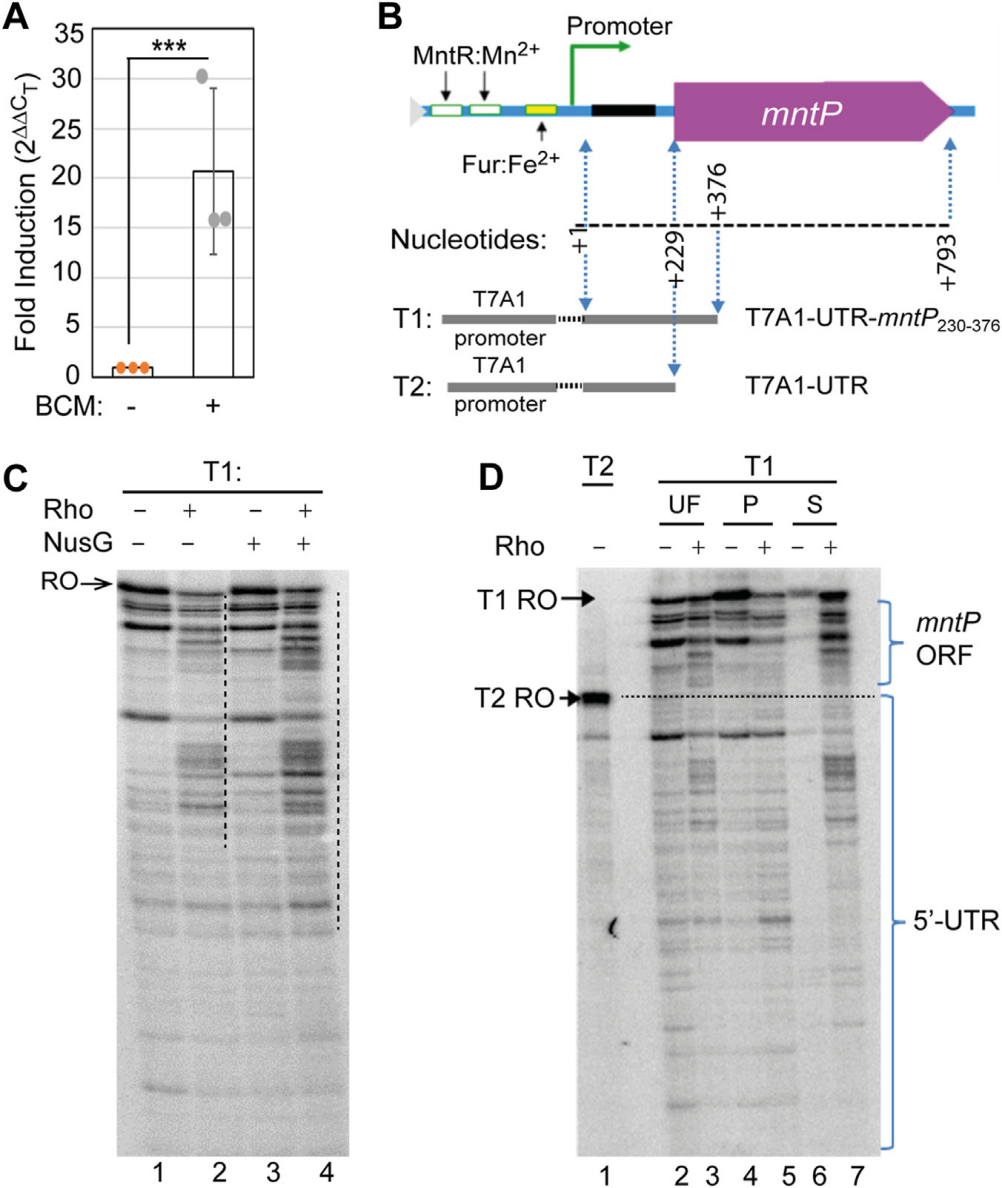
**Rho-dependent termination at the *mntP* gene locus.***A*, expression profile of *mntP* gene detected by Rt-PCR experiment. 100 μg/ml of BCM was used. The mean ± s.d. values from three independent experiments were plotted. ∗∗∗*p* < 0.001, unpaired *t* test. *B*, schematic showing the DNA region at the 5ˊ-UTR, *mntP* ORF, and T7A1 promoter to make T1 and T2 templates. *C*, Phosphor-imaging of 6% urea PAGE showing the *in vitro* transcription products on the T1 template in the presence or absence of Rho and NusG. RO marks the run-off product. The vertical *dotted lines* show the zone of Rho-dependent termination. *D*, phosphor-imaging of 6% urea PAGE shows unfractionated (UF), and fractionated samples (streptavidin pellet (P) and supernatant (S) fractions). The level of RO product using a shorter T2 template is shown.

Due to the presence of a sufficiently long 5′-UTR, we asked whether *mntP* gene expression is suppressed by transcription termination factor Rho. To test this possibility, we treated the growing *E. coli* wild-type (WT) cells with 100 μg/ml of bicyclomycin (BCM), a specific and potent antibiotic against Rho function (31, 40), and isolated the total cellular RNA. Using RT-PCR assay with the oligonucleotides specific for the open reading frame (ORF) of *mntP*, we found that BCM treatment upregulated the *mntP* gene by 20-fold (Fig. 1*A*). This observation suggests that the inactivation of Rho by BCM might block Rho-dependent termination at the 5′-UTR of *mntP*, causing an increased transcriptional read-through in the *mntP* ORF.

### Rho-dependent termination at the *mntP* gene locus demonstrated by *in vitro* transcription assays

In order to check whether the BCM-induced increase in *mntP* transcript is a direct result of Rho action, we made an *in vitro* transcription template (T1) fusing T7A1 promoter, which is an *E. coli* RNA polymerase-specific promoter, with 5′-UTR and 147 bp of *mntP* ORF by an overlapping PCR (Fig. 1*B*). A single round *in vitro* transcription assay was performed in the presence or absence of Rho. The transcription products were resolved by a urea-denaturing polyacrylamide gel electrophoresis (Urea-PAGE). Without Rho, the EC generated the full-length transcription run-off (RO) RNA at the end of templates. Some RNA transcripts shorter than the RO product were also generated, as the EC has an inherent tendency to pause at multiple locations during the transcription elongation process (Fig. 1*C*, lane 1). In the presence of Rho, the band intensity of the RO product declined (Fig. 1*C*, lane 2), but the intensity of the shorter RNA bands (Fig. 1*C*, lane 3), corresponding to some of the RNA polymerase pause bands (41) (Fig. 1*C*, lanes 1), were simultaneously increased. NusG is an additional transcription factor that assists the Rho-dependent termination process (29, 30, 31). We found that NusG enhanced the intensity of the shorter RNA bands in the presence of Rho (Fig. 1*C*, lane 4).

Although the intense bands of shorter transcripts that appeared in the presence of Rho are usually considered the putative Rho-dependent transcription termination products, they could be the mixture of the actual products of termination and transcriptional pausing of ECs (Fig. 1*D*). To separate the paused and terminated transcripts, we performed transcription assays, immobilizing the biotinylated template on streptavidin beads. The reaction products were separated into pellet and supernatant fractions. In the absence of Rho, most of the transcripts appeared in the pellet, while barely any transcripts were visible in the supernatant fraction in a urea-PAGE (Fig. 1*D*, lanes 4 and 6). However, when Rho was present, most of the shorter transcripts appeared in the supernatant than in the pellet fraction (Fig. 1*D*, lanes five and 7). The appearance of transcripts in the supernatant fraction indicates that Rho successfully terminated many transcripts before maturation. A shorter transcriptional RO product using a shorter DNA template, T7A1-UTR (T2), was generated as a marker (Fig. 1, *B* and *D*). The Rho-dependent transcription termination bands that were resolved below or above the position of the T2 RNA marker represent the termination that occurred in the 5′-UTR or initial 147 bp of *mntP* ORF, respectively (Fig. 1*D*). This data elucidates that Rho may terminate transcription at the 5′-UTR as well as at the ORF region of *mntP*.

### The activated riboswitch partially overcomes Rho-dependent termination

**Figure 2:**
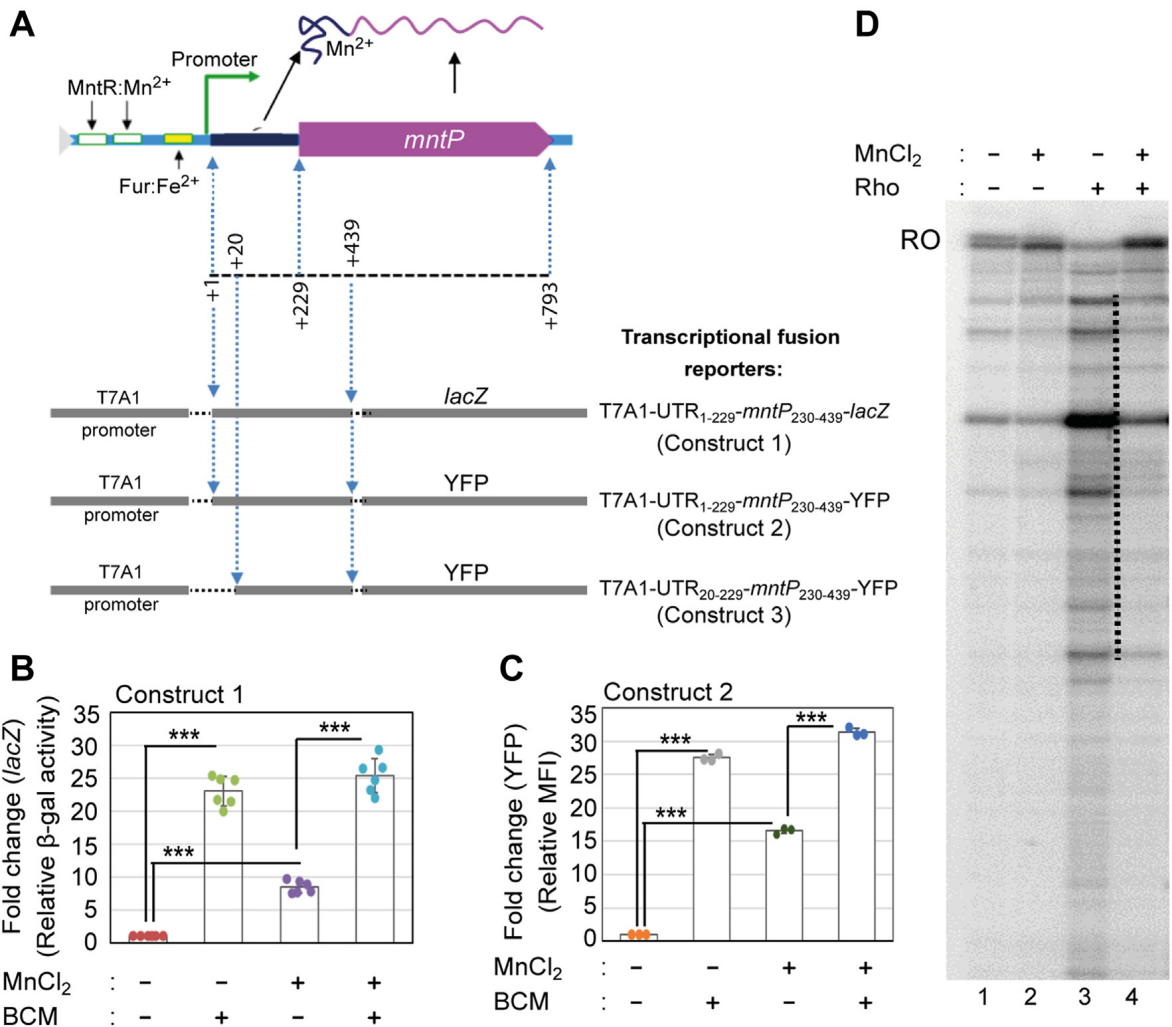
**The reporter assays to demonstrate that Rho prematurely terminates *mntP* expression.***A*, the schematic shows the DNA fragments from the 5ˊ-UTR and *mntP* ORF that have been fused with T7A1 promoter and YFP or lacZ reporters to make the reporter cassettes. *B*, the β-galactosidase activity from construct one in the presence or absence of 100 μg/ml BCM and 8 mM Mn are plotted. The calculated Miller unit for construct one was 545.5 ± 41. The values are mean ± s.d. from six independent experiments. ∗∗∗*p* < 0.001, unpaired *t* test. *C*, the estimated MFI of YFP from construct two are exhibited. The calculated values are mean ± s.d. from three independent experiments. ∗∗∗*p* < 0.001, unpaired *t* test. *D*, phosphor-imaging of *in vitro* transcription products on the T1 template in the presence or absence of Rho, and/or 100 μM Mn.

We then asked how the Rho-dependent termination affects the *mntP* riboswitch function or *vice versa*. The Mn activates the MntR regulator at the *mntP* promoter or *mntP*-riboswitch (6, 10). Therefore, to specifically check the riboswitch activation, we replaced the native promoter with the T7A1 constitutive promoter, which would not be regulated by Mn, to design three reporter strain constructs (construct 1, construct 2, and construct 3) (Fig. 2*A*). T7A1 promoter followed by the 5′-UTR and a portion of *mntP* ORF (encompasses +1 to +439 bp) was transcriptionally fused with either *lacZ* or YFP reporters. The reporter cassettes were integrated into the *E. coli* WT chromosome to generate the construct 1 and 2 reporter strains of *E. coli* (Fig. 2*A*). The design of the construct three will be described in a later section. Consistent with the RT-PCR trend (Fig. 1*A*), BCM treatment (100 μg/ml) enhanced the β-galactosidase activity and YFP fluorescence up to about 23 and 28-folds, in the construct one and 2, respectively (Fig. 2, *B* and *C*). This observation suggests that *in vivo* Rho-dependent termination at the *mntP* locus suppresses reporter expressions.

We also tested the effect of Mn on the expression profile of reporter constructs one and two. The MnCl_2_ (8 mM) shock (since 8 mM exogenous Mn causes sufficient toxicity in *E. coli* WT cells (18)) enhanced both β-galactosidase activity (9-folds) and YFP fluorescence (16-folds) (Fig. 2, *B* and *C*). BCM treatment to the Mn-fed *E. coli* cells further enhanced the β-galactosidase activity (25-folds) and YFP fluorescence (30-folds) that are comparable to the levels achieved by BCM treatment alone (Fig. 2, *B* and *C*). This observation indicates that while the Mn-mediated riboswitch activation partially upregulates the reporters, most of the transcripts still remain under regulation by Rho-dependent termination. In other words, Mn could partially inhibit Rho-dependent termination by activating *mntP* riboswitch to upregulate the reporter genes.

To address whether Mn directly interacts with Rho, thereby affecting Rho-dependent termination at the *mntP* riboswitch, we performed an *in vitro* transcription assay on the T1 template to test termination in a clean system. In the presence of Mn, longer transcripts representing transcription runoff re-appeared in a reaction containing termination factor Rho (Fig. 2*D*). However, unlike *in vivo* assay, here we detected a complete inhibition of Rho-dependent termination, possibly due to constraints in creating an identical *in vivo* situation in an *in vitro* assay. Nevertheless, the results, mainly the *in vivo* observation, support that *mntP* 5′-UTR serves as an Mn-dependent riboswitch by dialing down Rho-dependent termination.

### Free Mn does not influence Rho’s function

**Figure 3:**
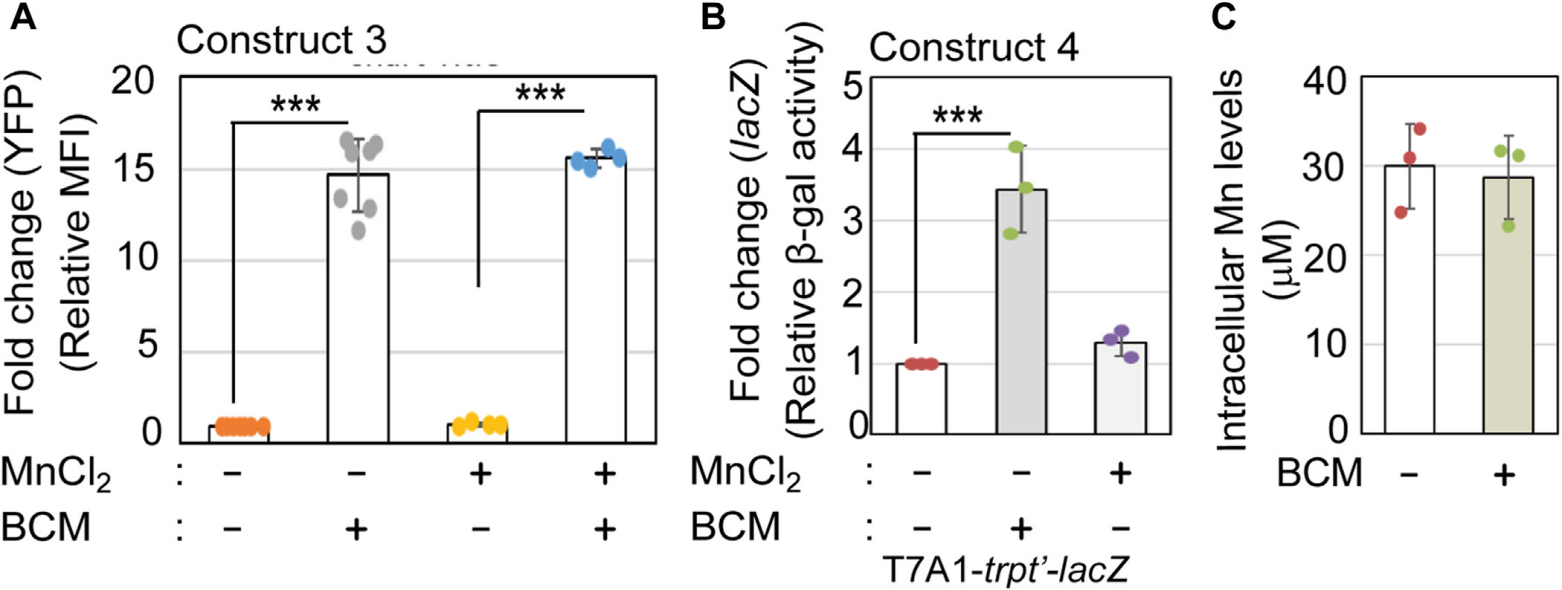
**Free Mn and Rho do not affect each other’s actions.***A*, the YFP fluorescence (MFI) values in construct three are plotted. 8 mM Mn or 100 μg/ml BCM were used, as indicated. The values are mean ± s.d. values from six independent experiments. ∗∗∗*p* < 0.001, unpaired *t* test. *B*, the relative β-galactosidase activities in construct four containing *trp t’* terminator are plotted. 8 mM Mn or 100 μg/ml BCM were used, as indicated. The calculated Miller unit for constructing four without any treatment was 4428 ± 902. The values are mean ± s.d. from three independent experiments. ∗∗∗*p* < 0.001, unpaired *t* test. *C*, the cellular Mn level in the presence or absence of 100 μg/ml BCM are plotted. The calculated mean ± s.d. values from three independent experiments were plotted. ∗∗∗*p* < 0.001, unpaired *t* test.

To test whether free Mn directly affects Rho’s termination function, we performed the following experiments. From the predicted structure of *mntP* riboswitch (10), we assumed that the absence of +1 to +19 nucleotides of the 5′-UTR would diminish the riboswitch activation while allowing the synthesis of a sufficiently long untranslated RNA for Rho-dependent termination. To test this possibility, we designed the third reporter strain (construct 3), where the T7A1 promoter followed by +20 to +439 bases of the *mntP* gene was fused with a YFP reporter and integrated into the WT strain (Fig. 2*A*). Indeed, Mn at 8 mM did not affect the mean fluorescence intensity (MFI) of YFP in construct 3 (Fig. 3*A*), suggesting that the absence of initial 19 residues in the 5′-UTR impair the formation of the Mn riboswitch. On the other hand, BCM alone, or BCM treatment to the Mn-fed construct three strain, increased the MFI of YFP to 15-fold (Fig. 2*D*). This observation indicates that the free Mn that could not form a complex with the truncated riboswitch has no effect on Rho function, and BCM-mediated inactivation of Rho directly allows transcription through the 5′-UTR to express YFP reporter.

To further ensure that Mn has no direct influence on the Rho function, we made another construct (construct 4), where the T7A1 promoter was fused with *trp t’* DNA that encodes a well-known Rho-termination site (24, 28, 42), and followed by a *lacZ* reporter. Using this construct, we show that 8 mM Mn did not affect the Rho-dependent termination process (Fig. 3*B*). Besides performing ICP-MS analyses, we determined that inactivating the Rho-dependent termination by BCM did not increase the cellular Mn levels (Fig. 3*C*). All these results suggest that Mn shock and Rho-dependent termination do not directly influence each other in affecting cellular physiology.

### *E. coli* is Mn-sensitive in the absence of the 5′-UTR

**Figure 4:**
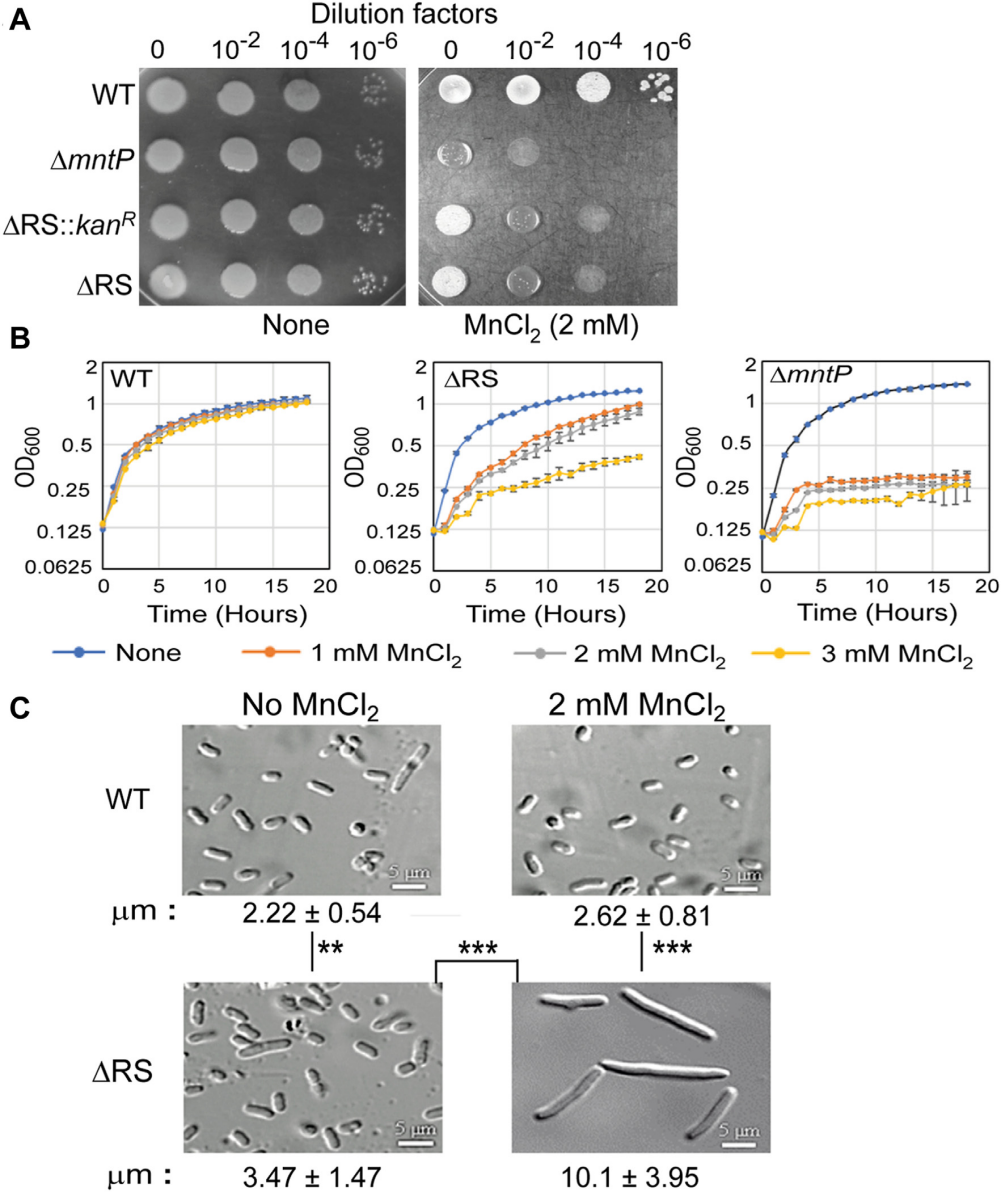
**Rho-dependent termination at 5′-UTR inhibits membrane protein toxicity.***A*, the spot assay shows that the riboswitch-deleted strains (Δ*RS::kan*^*R*^ and Δ*RS*) are Mn-sensitive. *B*, the WT, ΔRS, and Δ*mntP* strains were grown in the absence or the presence of different concentrations of Mn to compare the Mn sensitivity. *C*, confocal microscopy images show the cell filamentation of different strains of *E. coli*. The μM length of cells was calculated (mean ± s.d. values from 30-50 cells) and shown. ∗∗*p* < 0.01; ∗∗∗*p* < 0.001, unpaired *t* test. Fig. S1 exhibits a bigger area covering a greater number of cells.

We deleted the riboswitch (except +1 to +20 bp region) from the WT strain to get the riboswitch-deleted strains (Δ*RS*::*kan*^*R*^ and Δ*RS* strains) by the λ-red recombination system (43). Thus, we expected that the absence of 5′-UTR would defy Rho-dependent termination at the upstream of the *mntP* gene. This situation would lead to upregulation of MntP, causing a manganese-resistant phenotype in the riboswitch-deleted strains. To our surprise, the riboswitch-deleted strains unexpectedly exhibited Mn-sensitivity when grown on an LB-agar plate supplemented with 2 mM MnCl_2_ (Fig. 4*A*). The *E. coli* Δ*mntP* strain that fails to eject out excess Mn is also highly Mn-sensitive (7). Performing growth curve analyses, we find that 1 mM and 3 mM Mn caused roughly equivalent extents of growth defects to the Δ*mntP* and the Δ*RS* strains, respectively (Fig. 4*B*).

Performing confocal microscopy, we checked cellular morphology in the presence or absence of Mn. The WT strain exhibited a typical average cell length (2.22 ± 0.54 μM). In comparison, the Δ*RS* strain showed a significant increase in average cell length (3.47 ± 1.47 μM) in LB broth (Figs. 4*C* and S1). Under 2 mM Mn shock, the WT strain showed a significant but minor increase in average cell length (2.62 ± 0.81 μM), while the Δ*RS* strain was filamented (10.1 ± 3.95 μM) (Figs. 4*C* and S1). The filamentous phenotype of ΔRS strain was further analyzed by FM-4-64 and DAPI staining to show that the nucleoids were segregated but there was no septum formation in the filamentation (Fig. S2). Interestingly, the Δ*mntP* strain also exhibits Mn-dependent cell filamentation (7). In summary, the absence of *mntP* (in the Δ*mntP* strain) and the absence of the Rho-dependent termination/riboswitch function in the riboswitch-deleted strains both caused growth retardation and inhibition of cell division under Mn shock.

### The absence of the 5′-UTR overexpresses MntP under manganese stress

**Figure 5:**
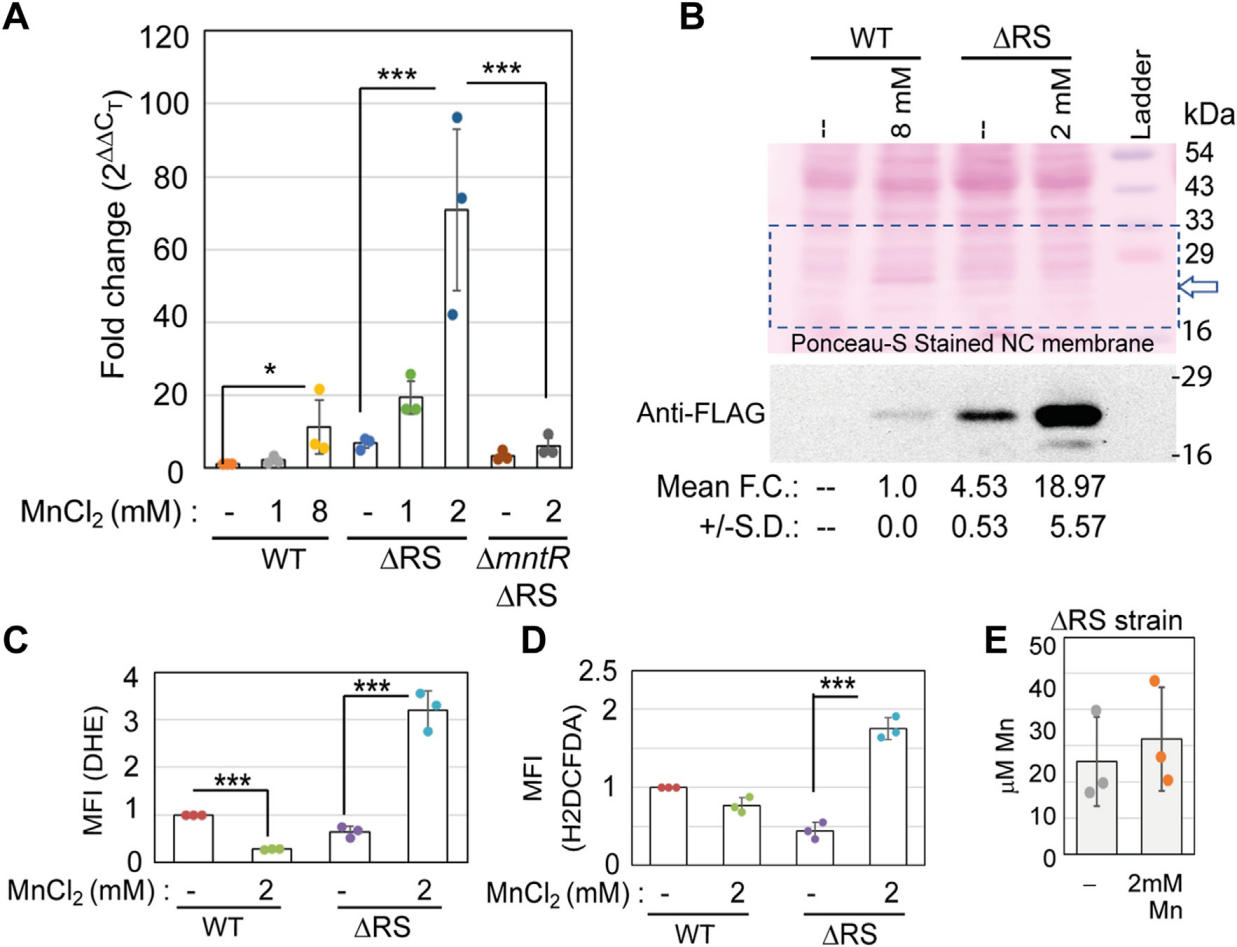
**Mn excessively upregulates *mntP* in the ΔRS strain.***A*, RT-PCR data show the *mntP* gene expression in the WT, Δ*RS*, and Δ*mntR* Δ*RS* strains in the presence or absence of Mn. The values are mean ± s.d. from three independent experiments. ∗*p* < 0.1; ∗∗*p* < 0.01, unpaired *t* test. *B*, Western blotting experiment shows overexpression of MntP-FLAG in the presence of Mn. The anti-FLAG signal remained below the detection level for untreated WT cells in our assay. Fold change (F.C.) values (mean ± s.d) were calculated using Image J software from three different Anti-FLAG blots and normalized against Mn-fed WT samples. Ponceau-S-stained membrane is shown to visualize the equal loading of the cellular proteins. *C*, the ROS-detecting probe, DHE, was used to show the fluorescence (MFI) from the WT and Δ*RS* strains in the presence or absence of Mn. The values are mean ± s.d. from three independent experiments. ∗∗∗*p* < 0.001, unpaired *t* test. *D*, the ROS detecting probe, H2DCFDA, was used to fluorescence (MFI) from the WT and Δ*RS* strain in the presence or absence of Mn. The values are mean ± s.d. from three independent experiments. ∗∗∗*p* < 0.001, unpaired *t* test. *E*, ICP MS analysis to check the intracellular levels of Mn (mean ± S.D) in the unfed and Mn-fed Δ*RS* strain.

The above observations raised a question of whether the Δ*RS* strain fails to express *mntP* and thereby mimics Δ*mntP*-like phenotypes. However, we noticed quite the opposite results when checking the expression of MntP by RT-PCR experiments. Mn at 1 mM, which is nontoxic to the WT cells (Fig. 4*A*), slightly enhanced the expression of *mntP* (2.5-folds) (Fig. 5*A*). When treated with a toxic dose (8 mM) of Mn (9), the WT cells exhibited about 11-fold upregulation of *mntP* (Fig. 5*A*). The Δ*RS* strain showed an inherently high level of *mntP* expression (7-folds) even in the absence of Mn shock (Fig. 5*A*). Strikingly, Mn at 1 mM and 2 mM levels increased this expression to about 19 and 70-fold, respectively (Fig. 5*A*). We assume that the activation of the MntR regulator by Mn upregulated the *mntP* in the Δ*RS* strain. Consistent with this assumption, unlike Δ*RS strain*, the Δ*mntR* Δ*RS* strain failed to upregulate *mntP* in the presence of 2 mM MnCl_2_ (Fig. 5*A*).

We also checked the cellular MntP protein level performing western blotting experiments. For this, we engineered the chromosome of WT and Δ*RS* strains by incorporating 3X-FLAG epitope-tag-coding sequence at the C-terminal end of *mntP* ORF. Using anti-FLAG antibody, we show that the exposure to the 8 mM Mn upregulated MntP in the WT strain compared to the untreated control (Fig. 5*B*). The Δ*RS* strain naturally exhibited a high basal level of MntP expression, suggesting that the absence of the Rho-dependent termination upregulated the MntP protein (Fig. 5*B*). The MntP level was excessively increased upon 2 mM Mn shock in the Δ*RS* strain (Fig. 5*B*).

The upregulation of *mntP* in the Mn-treated Δ*RS* strain was found to be associated with oxidative stress, as observed using dihydroethidium (DHE) and 2′,7′-dichlorodihydrofluorescein diacetate (H2DCFDA), two different ROS detecting chemicals (Fig. 5, *C* and *D*). This data is consistent with a previous observation that has shown that the *mntP* overexpression from a plasmid leads to oxidative stress in *E. coli* (44). Furthermore, we estimated the metal content of the cells to show that the Δ*RS* strain exhibited 25.6 ± 12 μM and 31.9 ± 14 μM intracellular Mn, respectively, in the absence or presence of 2 mM extracellular Mn, suggesting that MntP overexpression in the Mn-fed Δ*RS* strain did not deplete the cellular Mn levels (Fig. 5*E*).

### The overexpression of MntP in the ΔRS strain appears to be toxic

**Figure 6:**
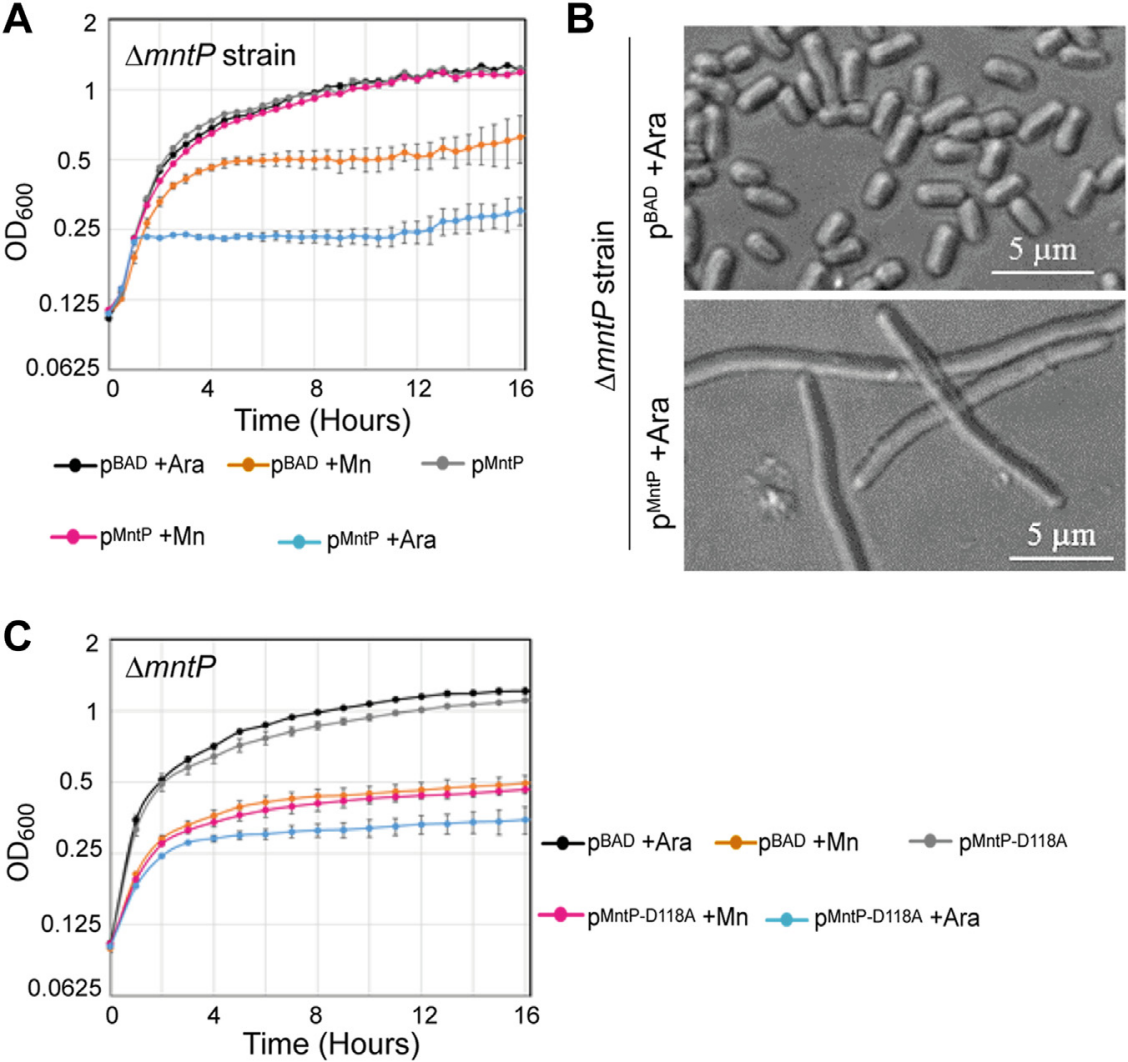
**Multicopy overexpression of MntP from an overexpressing plasmid is toxic.***A*, the growth curves show the growth effect when multicopy induction of MntP by arabinose (0.002%) was done in the Δ*mntP* strain. The calculated mean ± s.d. values from three independent experiments were plotted. *B*, the multicopy induction of MntP leads to cell filamentation. Fig. S3 shows bigger fields covering a greater number of cells. *C*, the growth curves assess the effect of multicopy induction of MntP^D118A^, which is a nonfunctional MntP, by arabinose (0.002%) in the Δ*mntP* cells. The calculated mean ± s.d. values from three independent experiments were plotted.

We observed growth defects and cell filamentation of the ΔRS strain under Mn shock (Fig. 4) despite the overexpression of MntP protein (Fig. 5). Therefore, we hypothesize that the Mn-sensitivity of the ΔRS strain may be the result of an excessive overexpression of MntP under Mn shock. To directly check whether *mntP* overexpression is toxic, we transformed the Mn-sensitive Δ*mntP* strain with an arabinose inducible pBAD-*mntP* expression vector. The multicopy leaky expression of *mntP* from the plasmid sufficiently rescued the Mn-sensitive growth phenotype of the Δ*mntP* strain (Fig. 6*A*). As a control, the Δ*mntP* strain harboring the pBAD empty vector did not rescue the Mn-dependent growth inhibition (Fig. 6*A*). However, when *mntP* expression from the pBAD-*mntP* vector was induced by 0.002% arabinose, the Δ*mntP* strain showed extreme growth retardation (Fig. 6*A*). Similar to the Mn-fed Δ*RS* strain (Fig. 4*C*), the overexpression of *mntP* from the plasmid led to cell filamentation (Figs. 6*B* and S3).

One may argue that an excessive Mn export by overexpressed MntP may lead to growth inhibition phenotype. To test this, we used a nonfunctional MntP mutant (pMntP^D118A^ transformed in the Δ*mntP* strain) (44) in the growth assay. The multicopy induction of MntP^D118A^ by arabinose (0.002%) did not rescue the growth defect (Fig. 6*C*). These data suggest that MntP overexpression but not its function is toxic to the cells.

### MntR-mediated transcriptional activation does not suppress Rho-dependent termination

**Figure 7:**
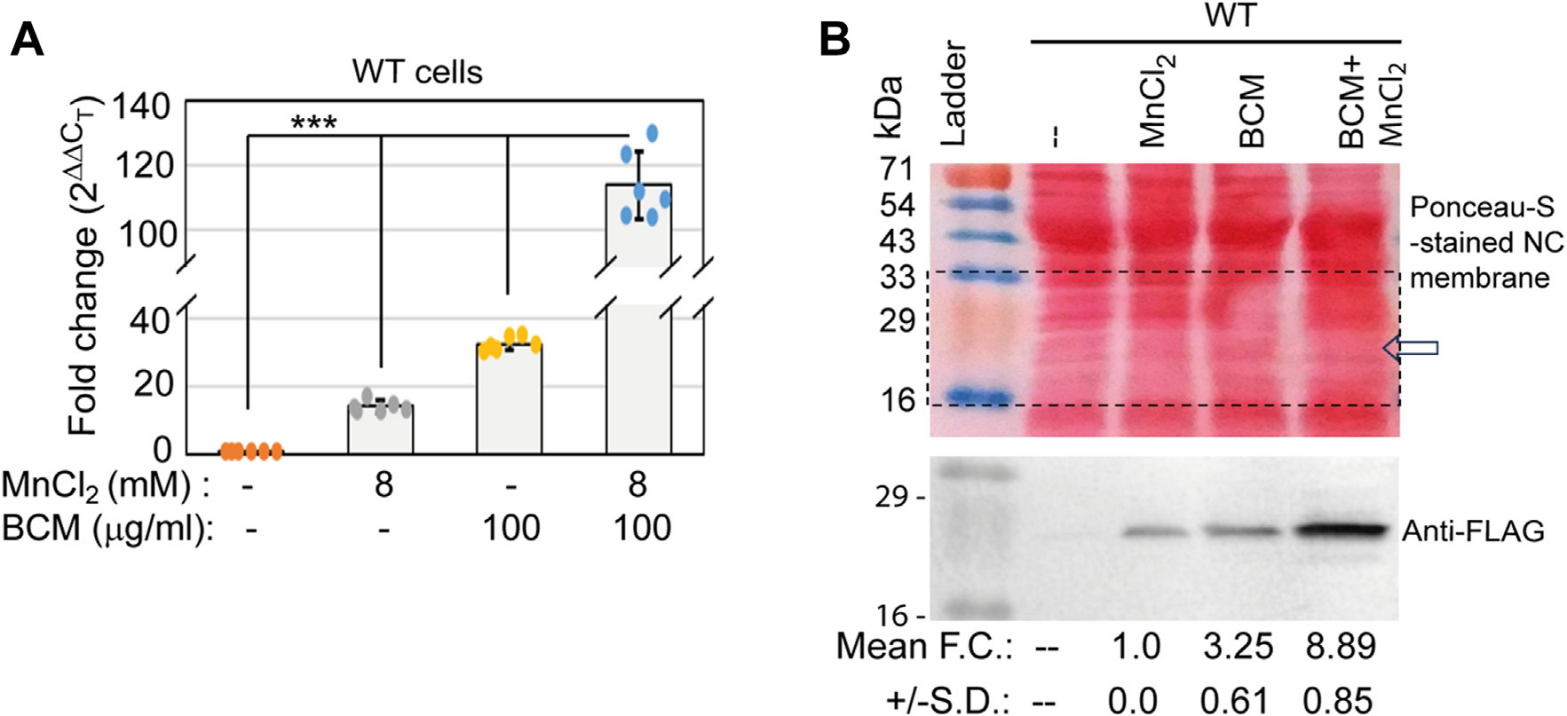
**MntR-mediated upregulation of *mntP* does not impact the Rho-dependent termination.***A*, the expression levels of *mntP* observed in the presence or absence of Mn (8 mM), BCM (100 μg/ml) or both. The fold change values (mean ± s.d.) from three independent experiments were plotted. ∗∗∗*p* < 0.001, unpaired *t* test. *B*, the Western blot experiments show the effect of Mn (8 mM), BCM (100 μg/ml) or both on MntP-FLAG expression. The anti-FLAG band intensities were calculated by Image J software. The band intensities were normalized and plotted. The anti-FLAG band remained below detection level in the untreated sample. The fold change values (mean ± s.d.) are from three independent blots.

We grew *E. coli* WT cells in the presence or absence of Mn and BCM to check whether MntR-mediated transcriptional upregulation can suppress Rho-dependent termination. Performing RT-PCR, we observed that MnCl_2_ and BCM separately upregulate *mntP* to about 15 and 30-folds (Fig. 7*A*). When the growing cells were treated with both MnCl_2_ and BCM together, a further increase in *mntP* expression (120-folds) was observed (Fig. 7*A*). We also performed western blotting experiments to show that Mn and BCM separately upregulated the MntP-FLAG expression, but the expression was highest when the growing cells were treated with MnCl_2_ and BCM together (Fig. 7*B*). These experiments suggest that *mntP* upregulation by MntR under Mn stress increases the level of *mntP* but this process does not influence the Rho-dependent termination. MntR and Rho act at two distinct phases of transcription, *viz.*, at the transcription initiation and elongation phases, respectively. The sites of their action are also spatially separated, *viz.*, MntP binds at the promoter DNA element, while Rho binds at the sufficiently long nascent mRNA to initiate a termination. Moreover, Rho is an abundant protein that remains in almost equimolar level that of RNA polymerase EC in the *E. coli* cells (27). Given these facts, it is quite possible that the Rho could catch up and terminate *mntP* transcripts at the elongation phase even when MntR upregulated the *mntP* gene at the initiation phase.

## Discussion

Our current study demonstrates that Rho is pivotal in terminating the transcription of *mntP* at the riboswitch. In the presence of Mn stress, the active form of the Mn^2+^:*mntP*-riboswitch switches on the translation (10), thereby partially evading Rho-dependent transcription termination and allowing MntP expression (Fig. 2, *B* and *C*). Thus, efflux of Mn by overexpressed MntP mitigates manganese stress.

A tight coupling between transcription and translation processes, *in vivo*, usually evades the Rho-mediated termination at the ORF (45, 46). In the absence of Mn, riboswitch's “switched off” conformation masks the SD site (10), and thereby the former would uncouple the transcription and translation processes. Thus, Rho-dependent termination suppresses roughly 24/25th parts of the reporter gene expression, while only 1/25th of parts could be expressed under the conditions examined (Fig. 2, *B* and *C*). In the presence of Mn shock, the alternative “switch on” conformation of riboswitch would evade Rho-dependent transcription termination in two different ways: one is by directly inhibiting Rho-binding to the RNA, and the other is by unmasking the SD, thereby facilitating transcription and translation coupling. Thus, under Mn stress, a modest increase (about 8/25th parts) in β-galactosidase activity was observed (Fig. 2, *B* and *C*).

While most riboswitches from *B. subtilis* affect intrinsic termination (11, 47), the *E. coli* riboswitches work primarily by modulating translation initiation (48). Consistently, the *mntP* riboswitch of *E. coli* works at the level of translation initiation (10). Interestingly, the majority of the *E. coli* riboswitches employ Rho-dependent transcription termination (35, 39). Rho is abundant and essential in *E. coli*; therefore, the scope of Rho-dependent termination is widespread in *E. coli* (27, 31, 49). On the other hand, Rho is scarce and dispensable in *B. subtilis*; therefore, most of the terminators are intrinsic in *B. subtilis* (50, 51). Accordingly, the binding of Mn to *yybP-ykoY* riboswitch precludes termination at the intrinsic terminator in upstream of *yybP* and in the leader region of *ykoY* (15). In *Lactococcus lactis*, another Gram-positive bacterium, a similar intrinsic terminator formation at the *yybP-ykoY* riboswitch region has been proposed at low Mn level (14). Thus, generalizing others’ (35, 39) and our current observations, it appears that the active riboswitches in *E. coli* defy the Rho-dependent termination by coupling transcription and translation processes and thereby allow gene expression. Whether this generalization has a broader scope for Gram-negative and Gram-positive bacteria needs to be deciphered.

Despite the riboswitch suppressing Rho-dependent termination, the bulk of total transcription (about 17/25th parts) was still prematurely terminated by Rho under the conditions examined (Fig. 2, *B* and *C*). We argued that most of the time, Rho quickly gets access to the nascent free RNA as it migrates with the EC (28) before Mn could form a complex with RNA to “switch on” the riboswitch under *in vivo* conditions. This argument explains how Rho-dependent termination at the *mntP*-riboswitch region ensures a substantially low level of MntP expression both in the presence or absence of Mn stress.

**Figure 8:**
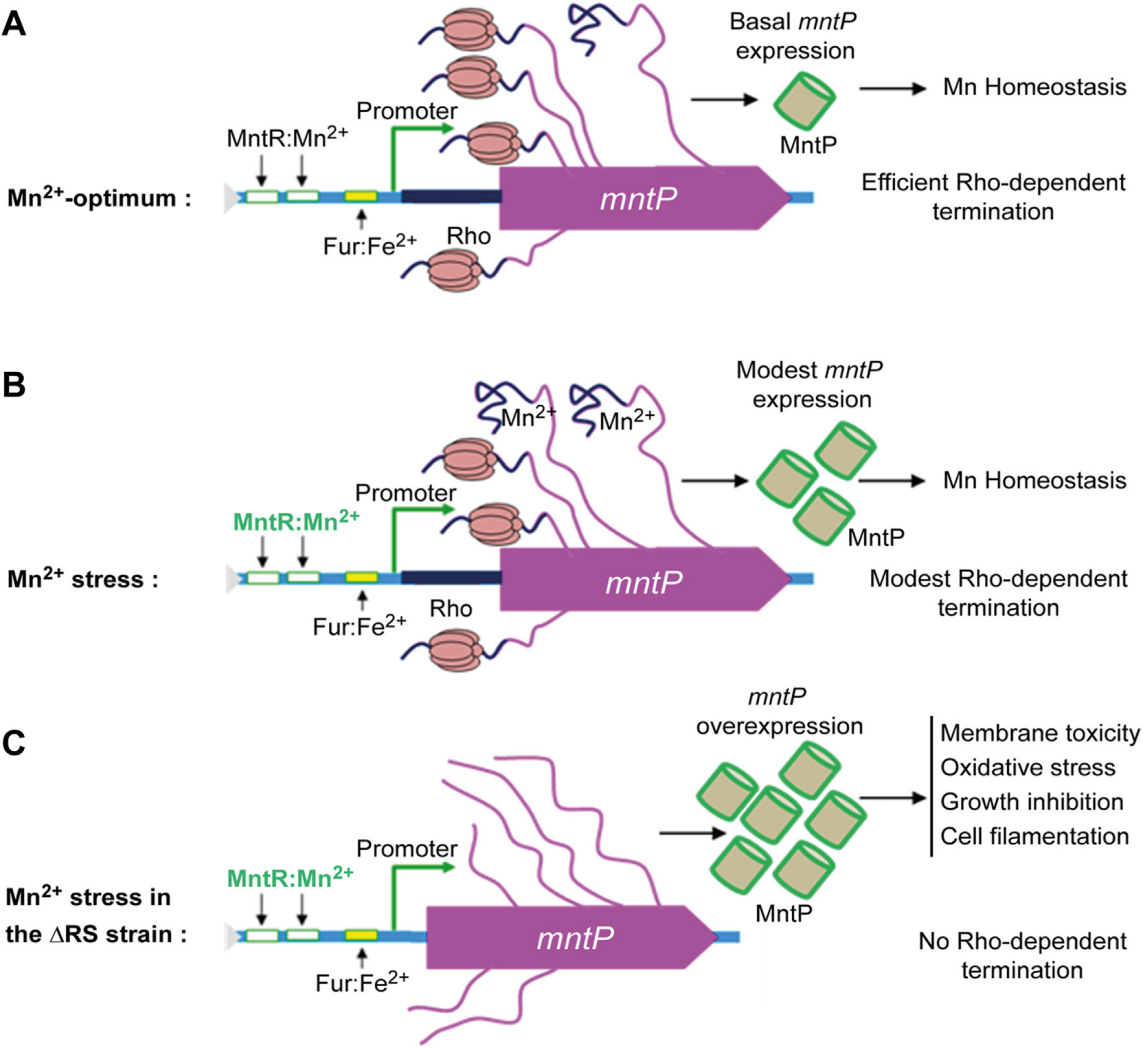
**Schematic showing the regulation of *mntP* expression by Rho and Mn.***A*, at optimum cellular Mn, Rho prematurely terminates transcripts at the *mntP* locus. Besides, MntR and riboswitch-mediated regulations also remain silent. All these aspects ensure a basal level of *mntP* expression for Mn homeostasis. *B*, under Mn stress, the active MntR: Mn^2+^ regulator, and the activated riboswitch, upregulate MntP to some extent. However, the majority of transcripts are still terminated by Rho prematurely, causing the modest level of MntP expression. *C*, in the case of the ΔRS strain, the absence of riboswitch RNA evades the Rho-dependent termination. Mn stress further activated the MntR regulator to upregulate MntP. Therefore, MntP was overexpressed uncontrollably, causing membrane protein toxicity.

We demonstrate that when Rho-dependent termination at the riboswitch RNA was defied in the Δ*RS* strain, an inherently high level of *mntP* expression was detected (Fig. 5, *A* and *B*). Mn stress increased this expression further (Fig. 5, *A* and *B*). However, why does Rho ensure low-level expression of MntP? We hypothesize that since MntP is an inner membrane protein, the Rho-dependent termination could suppress its expression to evade membrane protein toxicity. Generally, the overexpression of inner membrane proteins is highly toxic and leads to growth defects and cell filamentation (52, 53, 54, 55), as we also observed the same phenotype when MntP is overexpressed (Figs. 4 and 6). Therefore, we propose that the Rho-dependent transcription termination acts to suppress membrane protein toxicity by silencing MntP expression under Mn stress, as shown in the schematic (Fig. 8).

The mechanism of cytoplasmic membrane protein toxicity may be multifold. The membrane protein overexpression may exhaust the limited membrane space, leaving little room for others to be integrated, or may affect enzyme functions, especially those involved in oxidative phosphorylation/ATP synthesis (52, 53, 55). Besides, such overexpression may inhibit some crucial cellular functions by nonspecific electrostatic interaction with other membrane proteins (56). From our study, it appears that MntP overexpression possibly perturbed membrane biology and produced ROS, causing the slow growth and cell filamentation phenotypes (Figs. 4 and 6).

## Experimental procedures

### Bacterial strains, growth conditions, constructs, and plasmids

The wild-type BW25113 (WT) and the knockout mutant strains of *E. coli* from the KEIO collection used in this study are listed (Table 1). The mutant alleles were freshly introduced into the WT genome by P1 phage transduction. The FLP-recombination and P1 phage transduction methods were employed to generate double and triple mutants (43). The mutant alleles were subjected to PCR amplification and Sanger sequencing to authenticate the genotypes. Δ*RS*::*kan*^*R*^ and Δ*RS* strains were constructed by deleting the riboswitch region of *mntP* using pKD4 plasmid, RiboS1/S3 oligonucleotides, and λ-red recombination system (43).

**Table 1:** The list of strains and plasmids used in this work

Strains and plasmids	Genotype/Features	References
Strains		
BW25113	*Escherichia coli; rrnB3*Δ*lacZ4787hsdR514*Δ(*araBAD*) 567 Δ(*rhaBAD*)568 *rph-1*	(58)
Δ*mntP*	BW25113, Δ*mntP::kan*^*R*^	(58)
Δ*mntR*	BW25113, Δ*mntR::kan*^*R*^	(58)
Δ*mntH*	BW25113, Δ*mntH::kan*^*R*^	(58)
Δ*RS::kan*^*R*^	BW25113, Δ*RS::kan*^*R*^; (*mntP* riboswitch::*kan*^*R*^)	This study
Δ*RS*	BW25113, Δ*RS::scar*; (*kan*^*R*^ was removed)	This study
Δ*mntR* Δ*RS*	BW25113, Δ*mntR::kan*^*R*^, Δ*RS::scar*	This study
Δ*mntR* Δ*mntH* Δ*RS*	BW25113, Δ*mntR::kan*^*R*^, Δ*mntH::kan*^*R*^, Δ*RS::scar*	This study
BL21 (DE3)	F– *omp*T *hsdS*_*B*_ (r_B_–, m_B_–) *gal dcm* (DE3)	Lab collection
C43 (DE3)	A BL21 (DE3) derivative	(59)
Lemo (DE3)	BL21 (DE3) containing the Lemo System	(60)
Plasmids	Features	References
pET28a (+)	*kan*^*R*^; T7-promoter; IPTG inducible	Novagen
pET-*mntP*	*mntP* without a stop codon cloned in *NcoI* (blunted) and *HindIII* sites of pET28a (+) vector	This study
pAH125	*kan*^*R*^; LacZ reporter	(57)
pVenus	*kan*^*R*^; YFP reporter	(57)
pINT	*amp*^*R*^; λ-integrase	(57)
pKD4	*amp*^*R*^; *kan*^*R*^ cassette is flanked by FLP recombinase	(43)
pT7A1-*trp t’*	T7A1-trp t’ cassette cloned in pGEM-T (Promega)	This study
pBAD/*Myc*-His A	*amp*^*R*^; cloning vector	Invitrogen
pBAD-*mntP*	*mntP* cloned in *NcoI* (blunted) and *HindIII* sites of pBAD/*Myc*-His A vector	This study

*kan*^*R*^, kanamycin resistance; *amp*^*R*^, ampicillin resistance.

The DNA cassettes for the construct 1, 2, 3, and 4 strains were made by fusion PCRs using the appropriate pairs of oligonucleotides from Table S1. The DNA cassettes were cloned into pAH125 or pVenus vectors to get the desired transcriptional fusions (57). The cloned plasmids were then integrated into the genome of the WT strain using pINT helper plasmid and screened for single integrant, as described (57). To generate an *E. coli* strain with *mntP-*FLAG in the chromosome, we employed λ-red recombination system (43), as described in the supplementary materials.

The plasmid pBAD/*Myc*-His A (pBAD) (Invitrogen) was used for *mntP* cloning to generate a pBAD-*mntP* expression vector. PCR products were generated using GK1/GK2 primer pairs and digested with the *HindIII* enzyme. The vectors were digested with *NcoI* enzyme, blunted with Klenow enzyme, and further digested with *HindIII* enzyme. Digested vectors and PCR products were ligated and transformed to generate a pBAD-*mntP* expression vector. Site-directed mutagenesis was performed using DD149/DD150 primer pairs to generate pMntP^D118A^ vector from pBAD-*mntP*.

### Growth conditions

The bacterial growths were performed in buffered LB broth or media (pH 7.0) at 37 °C. Growth curve analyses were done using a Bioscreen C growth analyzer (Oy Growth Curves Ab Ltd), as described (9). For other assays, the overnight culture of *E. coli* WT cells was diluted 100-fold in the fresh LB medium with or without supplemented 8 mM Mn unless otherwise specified. The cultures were grown for 1.5 h at 37 °C till the O.D._600_ reached about 0.3. BCM (100 μg/ml) was added at that point and allowed to grow further for 2.5 h at 37 °C. The bacterial pellets were collected, and different assays were performed.

### Serial dilution and spot assays

Overnight cultures of the *E. coli* mutants were serially diluted, and 5 μl were spotted on LB-agar plates supplemented with or without 1 mM and 2 mM Mn to visualize the relative sensitivity while growing at 37 °C for 12 h 2 mM Mn nicely resolved the growth differences between the strains (Fig. 4*A*).

### RT-PCR analyses

The bacterial cell pellets were collected and quickly washed with 1X PBS. Total RNAs were isolated by TRIzol reagent and a bacterial RNA isolation Kit (Qiagen). DNase I treatment was done. The integrity and quality of the RNA was visualized in a 1% agarose gel. The RNA concentration was determined by a UV-1800 Shimandzu UV-spectrophotometer. 200 ng of RNA samples, primer pairs (Table S1), and GoTaq 1-Step RT-qPCR System (Promega) were used for RT-PCR assays. At least three independent assays were conducted. The change in fold expression in the treated samples was calculated by the ΔΔC_T_ method using *betB* mRNA RT-PCR as a control.

### In vitro transcription assay

The *in vitro* transcription using biotinylated T1 and T2 templates was performed, as described previously (21, 25). Briefly, 20 nM RNA polymerase, 40 nM templates (T1 or T2), 10 μM purified GTP and ATP, 25 μCi of [α-^32^P] CTP (BRIT), and 10 μM trinucleotide ApUpC (Oligos Etc.) were dissolved in 1X transcription buffer, TB (40 mM Tris-HCl, pH 7.9, 10 mM MgCl_2_, 50 mM KCl). The reaction mixture was incubated for 5 min at 37 °C to synthesize the initial 13 nucleotide long EC (EC_13_). 200 μM of nucleotide mixtures (of ATP, GTP, CTP, and UTP) were added to EC_13_ to synthesize RO products, incubating for 10 min at 37 °C. In the Rho-dependent termination assay, 100 nM Rho was added after synthesizing EC_13_ before adding the nucleotide mixtures. The reaction products were mixed with formamide loading dye and heat at 95 °C for 5 min to denature the RNA and other biomolecules. The denatured samples were resolved using a 6% urea-denaturing PAGE.

In another setup, the slurry of streptavidin beads equilibrated with 1X TB was mixed with EC_13_ and incubated for 5 min. Unbound substrates were removed by washing with 1X TB, and reactions were continued with or without Rho. After completion of the reaction, the supernatant portion was separated, and streptavidin beads/pellets were suspended in an equal volume of buffer. Both supernatant and pellet fractions were mixed with formamide loading dye, heated, and ran on a 6% urea-PAGE. The gels were exposed to the phosphor imager screen, and a Typhoon Scanner was used to capture the images.

### Western blotting

The western blotting experiments were performed using anti-FLAG polyclonal antibody (Invitrogen). The specificity of the antibody has been confirmed by Western blot experiment using protein extracts from WT cells containing chromosomal *mntP* or *mntP*-FLAG ORFs. The *E. coli* WT and ΔRS constructs containing chromosomal *mntP*-FLAG were grown in the absence or presence of Mn for 3 h. The B-PER reagent was used to lyse the cells, and protein contents were estimated by Bradford method. 40 μg of total cellular proteins were loaded on the SDS-PAGE. The proteins were transferred on a nitrocellulose membrane, stained with ponceau S to visualize the loading. Western blotting was done using HRP conjugated goat anti-rabbit secondary antibody and Immobilon Forte western HRP substrate (Millipore).

### β-galactosidase assay

For β-galactosidase assays, the bacterial strains (constructs 1, 3, and 4) were grown in the presence or absence of BCM and Mn, as described above. The cell pellets were collected and gently washed twice with Z-buffer (60 mM Na_2_HPO_4_, 40 mM NaH_2_PO_4_, 10 mM KCl, and 1 mM MgSO_4_), and the β-galactosidase assays were performed, as described (9).

### YFP reporter fluorescence assay

The overnight cultures were diluted 100-fold in LB broth and grown in the presence or absence of BCM and Mn, as stated above. The cell pellets were collected and washed with 1X phosphate buffer saline (PBS) and then resuspended in 1X PBS. The flow cytometry experiments were performed using a BD FACS accuri instrument for 0.1 million cells using an FL1 laser. The MFI values of three independent experiments with sd were plotted.

### Intracellular ROS detection

The overnight primary cultures were diluted 100 folds in LB broth and grown in the presence or absence of 2 mM Mn and 100 μg/ml BCM, as stated above. The cell pellet was washed with 1X PBS and divided into three different fractions, which had approximately equal numbers of cells. Of the three, one fraction was stained by 2 μM DHE, the other by 10 μM H_2_DCFDA for 1 hour, and the third fraction was dissolved in 1X PBS. The data was acquired using FACS accuri (BD) at FL1 laser for H_2_DCFDA and FL2 laser for DHE for 0.05 million cells. Mean fluorescence intensity (MFI) values obtained from three different experiments were plotted after deducting the background fluorescence values.

### Confocal microscopy analyses

The cell morphologies and filamentations were visualized using an in-house confocal Nikon confocal microscope. The overnight cultures of the WT and ΔRS *E. coli* strains were diluted 100-fold in fresh LB broth and were grown for 1.5 h at 37 °C till the O.D._600_ reached about 0.3. The cultures were further grown in the presence or absence of 2 mM MnCl_2_ for 2.5 h (for Fig. 4*C*). In case of Figure 6*B*, the arabinose was added from the beginning till 4 h of growth. The cell pellets were collected and washed with PBS. The cells were fixed with 4% formaldehyde and examined. The images were captured, and the relative cell lengths (50 cells for each condition) were measured by Image J. software. The mean and sd values were calculated.

### ICP MS analyses

The overnight culture of the WT and ΔRS strains was diluted 100-fold in fresh LB broth and was grown for 1.5 h at 37 °C till the O.D._600_ reached about 0.3. The WT culture was further grown in the presence or absence of 100 μg/ml of BCM for 2.5 h (for Fig. 3*C*). In the case of the ΔRS strain, the growth was continued for another 2.5 h in the presence or absence of 2 mM MnCl_2_ (for Fig. 5*E*). The cell pellets were collected, and ICP-MS analyses were done, as described (9).

## Data availability

All manuscript data are available in the manuscript files.

## Supporting information

This article contains Supporting information (43).

## Conflict of interest

The authors declare that they have no conflicts of interest with the contents of this article.
