# Supplementary material for: Rho and riboswitch-dependent regulations of mntP gene expression evade manganese and membrane toxicities
Source: J Biol Chem. 2024 Nov 5;300(12):107967. doi: 10.1016/j.jbc.2024.107967 (PMC11647493; doi:10.1016/j.jbc.2024.107967)
Supplement: Supporting information [file mmc1.pdf]

Supporting Information

Rho and riboswitch-dependent regulations of *mntP* expression evade manganese and membrane toxicities

Anand Prakash[1, ‡], Arunima Kalita[1, ‡], Kanika Bhardwaj[1, ‡], Rajesh Kumar Mishra[1], Debarghya Ghose[1], Gursharan Kaur[1], Neha Verma[1, 2], Bibhusita Pani[3], Evgeny Nudler[3,4] and Dipak Dutta[1, 2, *]

[1]Department of Molecular Biochemistry and Microbiology, CSIR Institute of Microbial Technology, Sector 39-A, Chandigarh 160036, India

[2]Academy of Scientific and Innovative Research (AcSIR), Ghaziabad, Uttar Pradesh 201002

[3]Department of Biochemistry and Molecular Pharmacology, New York University Grossman School of Medicine, New York, NY 10016, USA

[4]Howard Hughes Medical Institute, NYU Langone Health, New York, NY 10016, USA.
‡Equal contributions

[*] Correspondence: dutta@imtech.res.in

Present addresses for: Arunima Kalita, Department of Bacteriology, University of Wisconsin-Madison, 1550 Linden Drive Madison, Wisconsin 53706; Rajesh Kumar Mishra, Department of Biological Sciences, Indian Institute of Science Education and Research Mohali, Knowledge City, Sector 81, SAS Nagar 140306, India; Debarghya Ghose, Biotech Desk Pvt. Ltd, 3-5-22/2/A Krishnanagar, Secunderabad 500062, India.

Running title: Rho suppresses *mntP* expression

List of Materials

1. Supporting information Text
2. Figure S1, S2, S3 and S4
3. Table S1

Supporting information text

Construction of *E. coli* strains containing *mntP*-FLAG genotype

To generate *E. coli* strains with *mntP*-FLAG in the chromosome, we designed a synthetic DNA fragment containing homology 1 and homology 2 sites at the 3'- end of the *mntP* ORF from BiotechDesk, India. The homology-1 site, the 113-nucleotide long sequence from the 3'-end coding sequence of the *mntP* gene, was followed by a FLAG epitope coding sequence, Kan^R cassette of pKD4 vector, and homology-2 site. The synthetic DNA fragment was transformed in the WT and ΔRS strains and screened for kanamycin resistance. Finally, the Kan^R was removed by the FLP-recombinase system, as described (43). The Figure S4 shows the different sequence elements of *mntP*-FLAG synthetic DNA

Supplementary Figures

Figure S1.

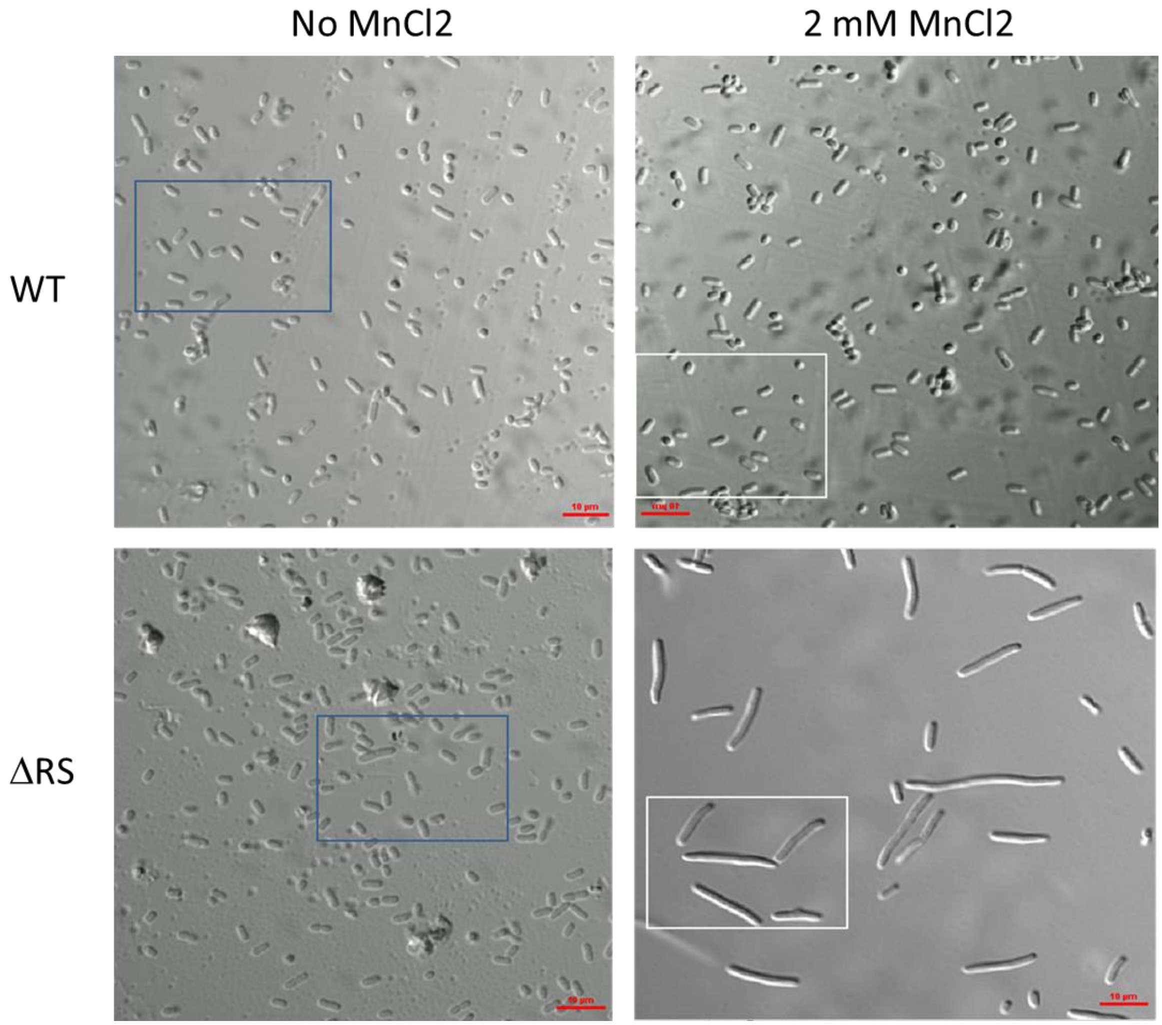

Figure S1. The cell morphologies judged under a confocal microscope when the WT and ΔRS strains were treated with Mn. The rectangular boxes in the panels are presented in Figure 4C.

Figure S2.

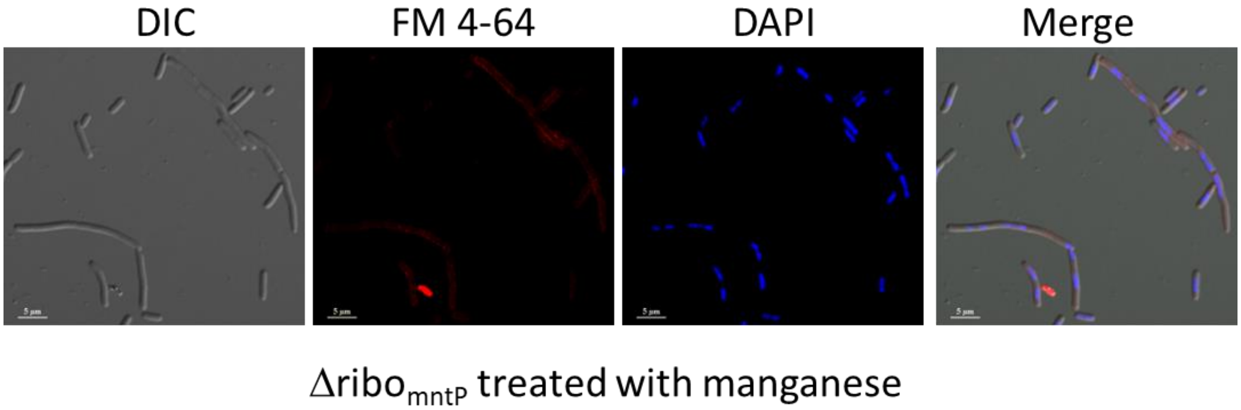

Figure S2. Mn-treated Δ*RS* cells were stained with FM-4-64 and DAPI to show that the filamentous cells had no septum, and discrete chromosomal DNA units were arranged throughout the filamented cells.

Figure S3.

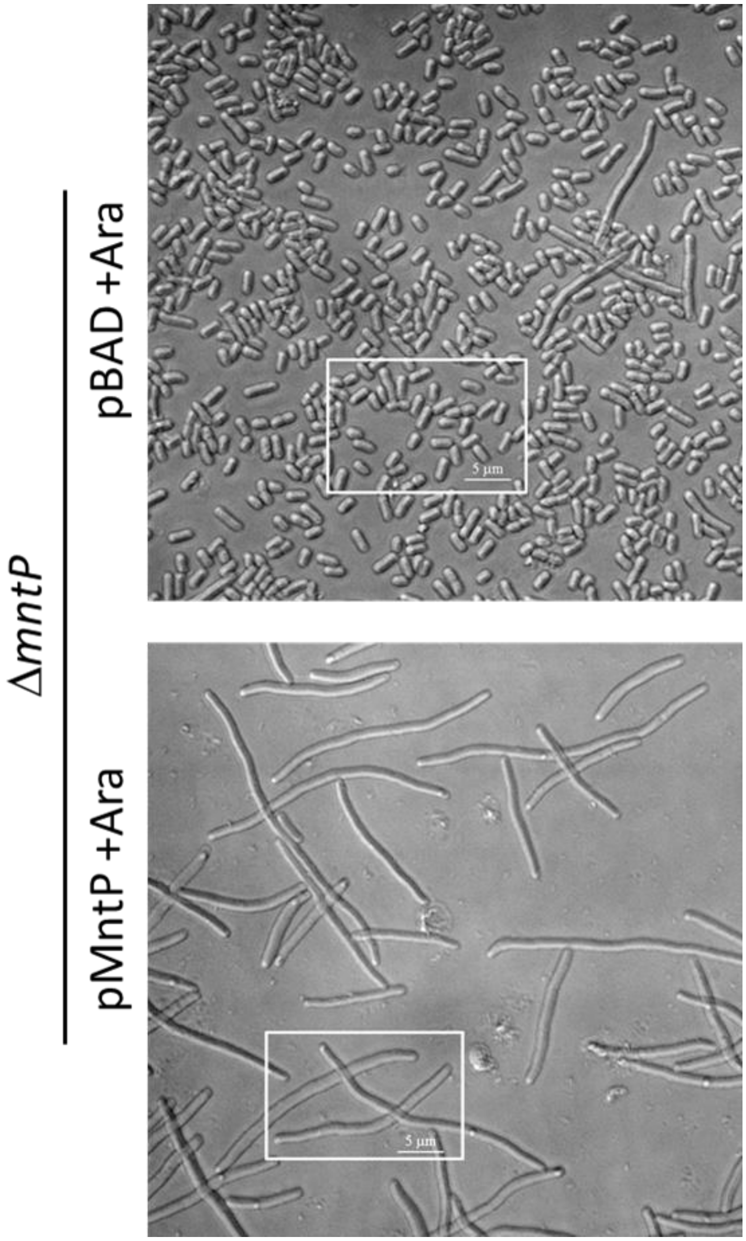

Figure S3. The cell morphologies judged under a confocal microscope with or without MntP overexpression. The rectangular boxes in the panels are presented in Figure 6B.

Figure S4.

A.

GGATGATGGTTGGTCGCTTTATCGGCTCAATTATTGGGAAAAAAGCGGAAATTCTCGGCGGGCTGGTGCTGATCGGCATCGGCGTC

CAGATCCTCTGGACGCACTTCCACGGTACTACTGCTAGCGAGAATTTGTATTTTCAGGGTGAGCTC**GACTACAAAGACCATGACGG**

TGATTATAAAGATCATGACATCGACTACAAGGATGACGATGACAAGTAGGTGTAGGCTGGAGCTGCTTCGAAGTTCCTATACTTTC

TAGAGAATAGGAACTTCGGAATAGGAACTTCAAGATCCCCTCACGCTGCCGCAAGCACTCAGGGCGCAAGGGCTGCTAAAGGAAGC

GGAACACGTAGAAAGCCAGTCCGCAGAAACGGTGCTGACCCCGGATGAATGTCAGCTACTGGGCTATCTGGACAAGGGAAAACGCA

AGCGCAAAGAGAAAGCAGGTAGCTTGCAGTGGGCTTACATGGCGATAGCTAGACTGGGCGGTTTTATGGACAGCAAGCGAACCGGA

ATTGCCAGCTGGGGCGCCCTCTGGTAAGGTTGGGAAGCCCTGCAAAGTAAACTGGATGGCTTTCTTGCCGCCAAGGATCTGATGGC

GCAGGGGATCAAGATCTGATCAAGAGACAGGATGAGGATCGTTTCGCATGATTGAACAAGATGGATTGCACGCAGGTTCTCCGGCC

GCTTGGGTGGAGAGGCTATTCGGCTATGACTGGGCACAACAGACAATCGGCTGCTCTGATGCCGCCGTGTTCCGGCTGTCAGCGCA

GGGGCGCCCGGTTCTTTTTGTCAAGACCGACCTGTCCGGTGCCCTGAATGAACTGCAGGACGAGGCAGCGCGGCTATCGTGGCTGG

CCACGACGGGCGTTCCTTGCGCAGCTGTGCTCGACGTTGTCACTGAAGCGGGAAGGGACTGGCTGCTATTGGGCGAAGTGCCGGGG

CAGGATCTCCTGTCATCTCACCTTGCTCCTGCCGAGAAAGTATCCATCATGGCTGATGCAATGCGGCGGCTGCATACGCTTGATCC

GGCTACCTGCCCATTCGACCACCAAGCGAAACATCGCATCGAGCGAGCACGTACTCGGATGGAAGCCGGTCTTGTCGATCAGGATG

ATCTGGACGAAGAGCATCAGGGGCTCGCGCCAGCCGAACTGTTCGCCAGGCTCAAGGCGCGCATGCCCGACGGCGAGGATCTCGTC

GTGACCCATGGCGATGCCTGCTTGCCGAATATCATGGTGGAAAATGGCCGCTTTTCTGGATTCATCGACTGTGGCCGGCTGGGTGT

GGCGGACCGCTATCAGGACATAGCGTTGGCTACCCGTGATATTGCTGAAGAGCTTGGCGGCGAATGGGCTGACCGCTTCCTCGTGC

TTTACGGTATCGCCGCTCCCGATTCGCAGCGCATCGCCTTCTATCGCCTTCTTGACGAGTTCTTCTGAGCGGGACTCTGGGGTTCG

AAATGACCGACCAAGCGACGCCCAACCTGCCATCACGAGATTTCGATTCCACCGCCGCCTTCTATGAAAGGTTGGGCTTCGGAATC

GTTTTCCGGGACGCCGGCTGGATGATCCTCCAGCGCGGGGATCTCATGCTGGAGTTCTTCGCCCACCCCAGCTTCAAAAGCGCTCT

GAAGTTCCTATACTTTCTAGAGAATAGGAACTTCGGAATAGGAACTAAGGAGGATATTCATATTTTTGCTGCCAGTGTTTGCCAG

ACTTCTGGCTTCGCACGCCAGGCAAACGGCGTCATCTGCAATAATGCGACGGCTTCATCACCGCGAAGAC

B.

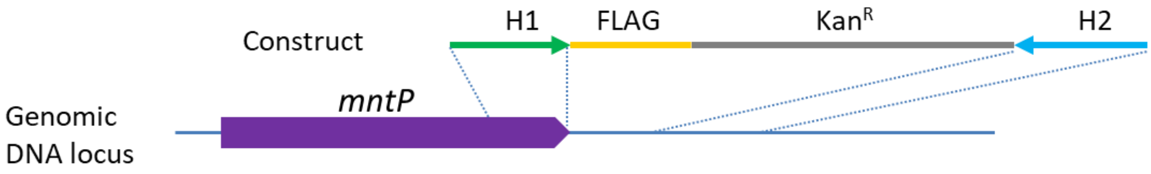

Figure S4. A. The synthetic DNA sequence showing green highlighted Homology 1 site taken from the 3'-end of *mntP ORF*, yellow highlighted FLAG epitope-containing coding sequence, grey highlighted Kan^R cassette flanked by FRT sites from pKD4 vector, cyan highlighted Homology 2 site taken from the noncoding DNA downstream of the *mntP ORF***. B.** The schematic indicates the synthetic DNA construct and it different parts. The integration sites of the synthetic DNA in the genomic DNA are marked.

Table S1. List of oligonucleotides used in this study

Oligonucleotides	Sequence	Description
T7A1_F	CACTGAGGTACCTCCAGATCCCG AAAATTTATCAA	T7A1 promoter Forward primer With *KpnI* site.
DD6	GAACTCCAGCGGTTGCTCCCTCT CGA	T7A1 promoter Reverse primer
AK 1 mntP_T7A1	AGCAACCGCTGGAGTTCGCCCTC ATTTGGGGAGTAG	*mntP* riboswitch Forward primer with T7A1-reverse primer overhang.
D175	AGCAACCGCTGGAGTTCCCGA TTTCCAGATTCCG	*mntP* riboswitch Forward primer with T7A1-reverse primer overhang
mn70rev	GTCAGTGAATTCTTAGTGGTT CCATTCAAGGAC	Reverse primer with *EcoRI* restriction site
RiboS1	TATATTCTCAAAATATGTTAA GGTTGCGCCCTCATTTGGGGA GTAGCCGTGTAGGCTGGAGCT GCTTCG	Forward primer for *mntP* riboswitch deletion
RiboS3	CATCCATCGACATACCAAACG CAAGAAGAACAGTAGCAGTG ATATTCATATGAATATCCTCC TTAG	Reverse primer for *mntP* riboswitch deletion.
D174	GCTTAGGCTATATTACCTC	Forward primer to check riboswitch deletion by PCR and sequencing
*mntP*F	ATCACGGCTTGTTGTTCATG	Forward primer for checking *mntP* ORF integrity by sequencing
*mntP*R	CAGAAGTCTGGCAAACACTG	Reverse primer for checking *mntP* ORF integrity by sequencing
T7A1P	TCCAGATCCCGAAAATTTATC AA	T7A1 promoter forward PCR primer for in vitro transcription template T1 and T2 preparation

DD144	CAGGGTTTCGACGGCAGCAA AAATAAGGCCGGTTC	Reverse PCR primer for in vitro transcription template T1 preparation
D176	CATGACAATGTCCTGACC	Reverse PCR primer for in vitro transcription template T2 preparation
GK1	AATATCACTGCTACTG	*mntP* ORF forward primer to clone in pBAD vector
GK2	ACAAGTAAGCTTTAACCGTGG AAGTGCGTC	*mntP* ORF reverse primer with stop codon to clone in pBAD vector
DD149	GATTGCCACCAGCCTGGCTGC CATGGCTGTGG	SDM primer 1 to generate $pMntP^{D118A}$ vector from pBAD-*mntP*
DD150	CCACAGCCATGGCAGCCAGG CTGGTGGCAATC	SDM primer 2 to generate $pMntP^{D118A}$ vector from pBAD-*mntP*
KK2	AGCAACCGCTGGAGTTCGTCG AACGTCAACTTAC	*trp t'* forward primer with DD6 overhangs
KK3	GTATGGAATTCCCCGTTGCGT TGCATTGTTTC	*trp t'* reverse primer with *EcoRI* site
mntPF-RT:	CGTCGACACGGTTTCTG	*mntP* forward primer for qPCR
mntPR-RT:	AGCGACCAACCATCATCCCT	*mntP* reverse primer for qPCR
betBF	AACTTCTTCAGCTCCGGTCA	*betB* forward primer for qPCR
betBR	GCCGAAGTTAGTTTGCGGAT	*betB* reverse primer for qPCR
